# A practical protocol for measurements of spinal cord functional connectivity

**DOI:** 10.1038/s41598-018-34841-6

**Published:** 2018-11-08

**Authors:** Robert L. Barry, Benjamin N. Conrad, Seth A. Smith, John C. Gore

**Affiliations:** 1 Athinoula A. Martinos Center for Biomedical Imaging, Department of Radiology, Massachusetts General Hospital, Charlestown, Massachusetts USA; 2 Department of Radiology, Harvard Medical School, Boston, Massachusetts USA; 3 Vanderbilt University Institute of Imaging Science, Vanderbilt University Medical Center, Nashville, Tennessee USA; 4 Neuroscience Graduate Program, Vanderbilt University Medical Center, Nashville, Tennessee USA; 5 Department of Radiology and Radiological Sciences, Vanderbilt University Medical Center, Nashville, Tennessee USA; 6 Department of Biomedical Engineering, Vanderbilt University, Nashville, Tennessee USA

## Abstract

Resting state functional magnetic resonance imaging (fMRI) has been used to study human brain function for over two decades, but only recently has this technique been successfully translated to the human spinal cord. The spinal cord is structurally and functionally unique, so resting state fMRI methods developed and optimized for the brain may not be appropriate when applied to the cord. This report therefore investigates the relative impact of different acquisition and processing choices (including run length, echo time, and bandpass filter width) on the detectability of resting state spinal cord networks at 3T. Our results suggest that frequencies beyond 0.08 Hz should be included in resting state analyses, a run length of ~8–12 mins is appropriate for reliable detection of the ventral (motor) network, and longer echo times – yet still shorter than values typically used for fMRI in the brain – may increase the detectability of the dorsal (sensory) network. Further studies are required to more fully understand and interpret the nature of resting state spinal cord networks in health and in disease, and the protocols described in this report are designed to assist such studies.

## Introduction

For over two decades, functional magnetic resonance imaging (fMRI) studies of resting state connectivity in the brain have provided unparalleled insight into the functional architecture of the central nervous system in health^1,2^ and disease^3^. While the vast majority of fMRI studies have been restricted to the brain and its crucial roles, a small but growing number of papers have explored fMRI in the healthy human spinal cord^4^. Until very recently, however, spinal cord fMRI studies have been task-based, primarily focusing on the manifestation of evoked signal changes due to sensorimotor processing^5,6^ and pain^7–9^, but also on changes due to arousal^10^ and global vasodilation^11^. In 2014, we reported our observations of correlated blood oxygenation level dependent (BOLD) “resting state” fluctuations within spinal cord gray matter at 7 Tesla^12^. The observations of reproducible correlations between ventral horns and between dorsal horns were also shown to be reproducible within-subject^13^, and have been reported in other species^14,15^, providing further evidence for the existence of functional networks in a resting state within the cord.

Our first studies were performed at 7T to benefit from the increased BOLD contrast-to-noise ratio (CNR) at higher fields^16,17^, and non-human primate studies were similarly performed in parallel at 9.4 Tesla^14^, but investigations by other groups subsequently confirmed that these networks are detectable at 3 Tesla^18–20^. Recent studies have shown remarkable agreement between 3T and 7T in the connectivities of both bilateral motor and sensory networks^21–24^. A recent study showed that these networks may be modulated by thermal stimulation^23^, and the amplitude of low-frequency fluctuations^25^ have been shown to differ between healthy subjects and patients with cervical spondylotic myelopathy^26^ and fibromyalgia^24^. The reproducibility of detecting functional networks at different field strengths using different data acquisition, processing, and analysis methods, as well as in other species^14,15^, suggests that functional connectivity metrics within the cord could be used as potential biomarkers of spinal cord injury or disease^27^. As a step toward more widespread practical applications of this approach, the impact of various acquisition, processing, and analysis methods on the detection of these spinal cord networks needs to be established.

Previous studies of fMRI in the brain have considered, for example, the effects of echo time (TE)^17,28^ and flip angle^29^ on BOLD CNR, showing that functional CNR is weakly dependent upon TE over a broad range, and nearly invariant of flip angle. Similarly, while resting state runs are commonly 8 mins or less, a recent study showed that increasing run length to 9–12 mins significantly increases test-retest reliability^30^. Finally, while most resting state studies consider only a low-frequency bandwidth between 0.01 and ~0.1 Hz, a growing number of reports suggest that the inclusion of higher frequencies, for example, up to 0.5 Hz^31^, 0.75 Hz^32^, and potentially even 3.7 Hz^33^, may increase the detectability of functional networks. Two previous studies provide strong evidence for retaining temporal frequencies above 0.1 Hz in the spinal cord^13,21^, and our current study builds upon these reports.

Resting state fMRI in the spinal cord is similarly influenced by technical acquisition factors and post-processing choices, but the relative impacts of individual parameters have not previously been systematically evaluated. For example, the presence of susceptibility gradients and motion are different in the spine compared to the brain^34,35^, and have led to our use of 3D gradient echo acquisition sequences with reduced distortion rather than conventional echo planar imaging^12^. Thus, this paper aims to investigate the impact of several acquisition and processing choices on the detectability of resting state spinal cord networks at 3T. One study evaluated two 20-min acquisitions with three bandpass filter ranges, and a second investigated the effects of increasing TE on functional image quality and network detectability. While these studies use 3D gradient echo acquisition sequences to mitigate distortions, future optimization studies may similarly consider the use of more traditional 2D echo planar imaging (EPI) sequences.

## Results

**Figure 1:**
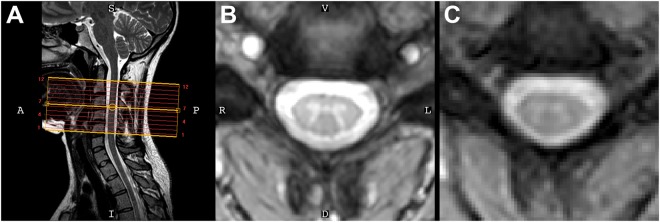
(**A**) Mid-sagittal slice from a healthy volunteer [S = superior, I = inferior, A = anterior, P = posterior]. (**B**) High-resolution anatomical image at C4 (acquired voxel size = 0.65 × 0.65 × 5 mm^3^, interpolated voxel size = 0.29 × 0.29 × 5 mm^3^) [V = ventral, D = dorsal, R = right, L = left]. (**C**) Functional image shows minimal distortions and excellent conspicuity between cerebrospinal fluid and the cord (acquired voxel size = 1 × 1 × 5 mm^3^, and interpolated to match the resolution of the anatomical image). The orientation shown in (**B**) is used for all axial images throughout this paper and the supplementary material.

Figure 1 presents imaging data from one volunteer. Axial slices were planned perpendicular to the cord to obtain coverage of vertebrae C2 to C5 (Fig. 1A). High-resolution (0.65 × 0.65 mm^2^) averaged multi-echo gradient echo axial images (Fig. 1B) clearly show the characteristic gray matter butterfly, and a similar pattern is observed in T_2_*-weighted fMRI data (Fig. 1C).

### Effects of scan length

**Figure 2:**
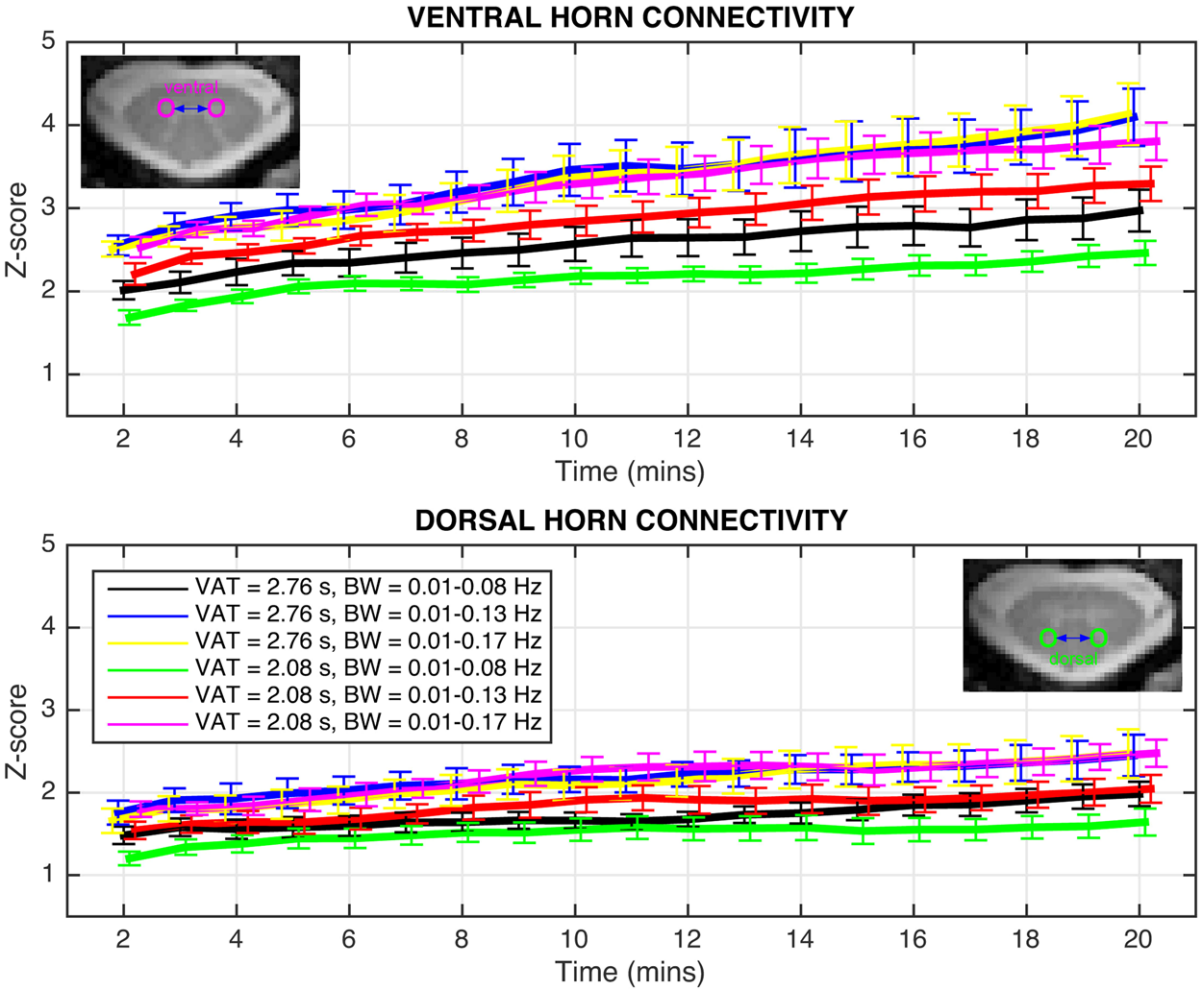
Functional connectivity between ventral horns (top) and dorsal horns (bottom) for both acquisition sequences and three bandpass filter ranges for time series between 2 and 20 mins. Only points at every minute are displayed and analyzed, and, for clarity, the curves are temporally offset from one another slightly to better visualize the error bars. Error bars represents standard error of the mean across subjects. In the bottom panel, the figure legend relating color to volume acquisition time (VAT) and bandwidth (BW) refers to curves in both top and bottom panels.

The effects of scan length are presented in Fig. 2 for both acquisition sequences and three bandpass filter ranges (bandwidth; BW = 0.01–0.08 Hz, 0.01–0.13 Hz, and 0.01–0.17 Hz) for truncated time series between 2 and 20 mins. For “full-k” data, gray matter temporal signal-to-noise ratio (TSNR) = 19.4 ± 3.1 after functional-to-anatomical registration, and then 22.6 ± 4.4 after data-driven de-noising. Similarly, for “part-k” data, TSNR = 17.7 ± 3.1 after registration and then 20.0 ± 4.0 after de-noising. In each plot, the only difference between the black, blue, and yellow curves, and the green, red, and magenta curves, respectively, is the frequency width of the filter. Two main observations may be made about these data. Firstly, ventral horn correlations are consistently higher than dorsal horn correlations, and have a larger dynamic range between the minimum and maximum run lengths. Secondly, the primary factor influencing detectability (i.e., *z*-scores) of both ventral and dorsal networks is not run length but rather the BW of the bandpass filter. The results obtained from BW = 0.01–0.08 Hz, which was the first frequency range considered^1^ and is widely used in resting state brain fMRI, are the lowest for both networks for either acquisition sequence (black and green curves). Extending the upper frequency to 0.13 Hz (blue and red curves) or 0.17 Hz (yellow and magenta curves) shifts the *z*-scores upward relative to the respective black and green curves.

If we consider an 8-min resting state run, which is commonly performed in studies of the brain, then statistical comparisons may be made at this time point and between later time points. For ventral horn connectivity at *t* = 8 mins, *z*-scores with [volume acquisition time; VAT = 2.76 s and BW = 0.01–0.13 Hz] (blue curve) are higher (unpaired, *p* < 0.05) than [VAT = 2.08 s and BW = 0.01–0.08 Hz] (green curve). No significant differences are observed between the three BW options for VAT = 2.76 s. For VAT = 2.08 s, BW = 0.01–0.17 Hz (magenta curve) resulted in higher *z*-scores (paired, *p* < 0.01) than BW = 0.01–0.08 Hz (green curve).

For dorsal horn connectivity, no significant differences are observed between the six acquisition strategies at *t* = 8 mins. No significant differences are observed between the three BW options for VAT = 2.76 s. For VAT = 2.08 s, BW = 0.01–0.13 Hz (red curve) and BW = 0.01–0.17 Hz (magenta curve) both resulted in higher *z*-scores (paired, *p* < 0.05) than BW = 0.01–0.08 Hz (green curve).

Based upon these results, the acquisition and filtering strategy [VAT = 2.08 s and BW = 0.01–0.17 Hz] (magenta curve) is selected to identify the point at which a longer run length produces significantly higher *z*-scores. For ventral horn connectivity, comparisons between *z*-scores at *t* = 8 mins and *t* = 9, 10, 11, and 12 mins, respectively, show that *z*-scores are significantly higher at both *t* = 11 mins (paired, *p* < 0.05) and *t* = 12 mins (paired, *p* < 0.001). For dorsal horn connectivity, *z*-scores are not significantly different between *t* = 8 mins and *t* = 9, 10, 11, or 12 mins. A subsequent comparison between *z*-scores at *t* = 8 mins and longer runs from *t* = 13 mins to *t* = 20 mins revealed significant differences only starting at *t* = 18 mins (paired, *p* < 0.05). Additional group analyses measuring the reproducibility (i.e., intraclass correlation coefficient^36^) of *z*-scores across time and at *t* = 10 mins are presented in Supp. Figs S3 and S4, respectively. Finally, because *z*-scores are calculated herein as the product of the inverse hyperbolic tangent of the Pearson correlation coefficient and a term related to the estimated degrees of freedom after correction for first-order auto-correlation (described in Methods), Supp. Figs S5 and S6, respectively, present the Pearson correlation coefficients and degrees of freedom correction factors for these group analyses^37^. A summary of all statistically significant comparisons is presented in Supp. Table S1.

**Figure 3:**
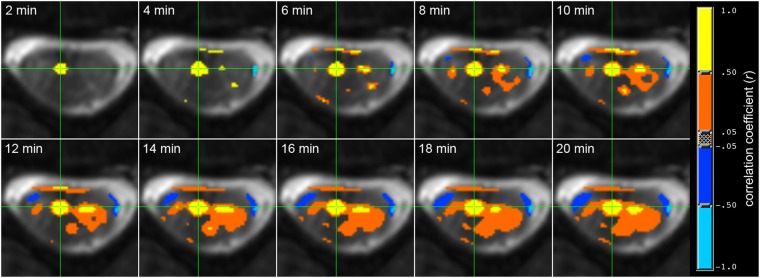
Functional connectivity between ventral horns for increasing run length between *t* = 2 and *t* = 20 mins. Analysis performed in AFNI^50^ using the ‘InstaCorr’ function with a fixed statistical threshold of *p* < 0.001. Yellow denotes high temporal correlation (*r* > 0.50) with the single voxel time course at the green crosshair, and blue represents anti-correlations. The motor network becomes detectable at *t* = 6 mins and stable when *t* ≥ ~12 mins.

Figure 3 visualizes a seed-based ventral horn connectivity analysis from one subject for increasing run length from *t* = 2 to *t* = 20 mins. Consistent with the preceding group results, the spinal cord motor network for this subject becomes detectable at *t* = 6–10 mins and stable when *t* ≥ 12 mins.

### Effects of echo time

**Figure 4:**
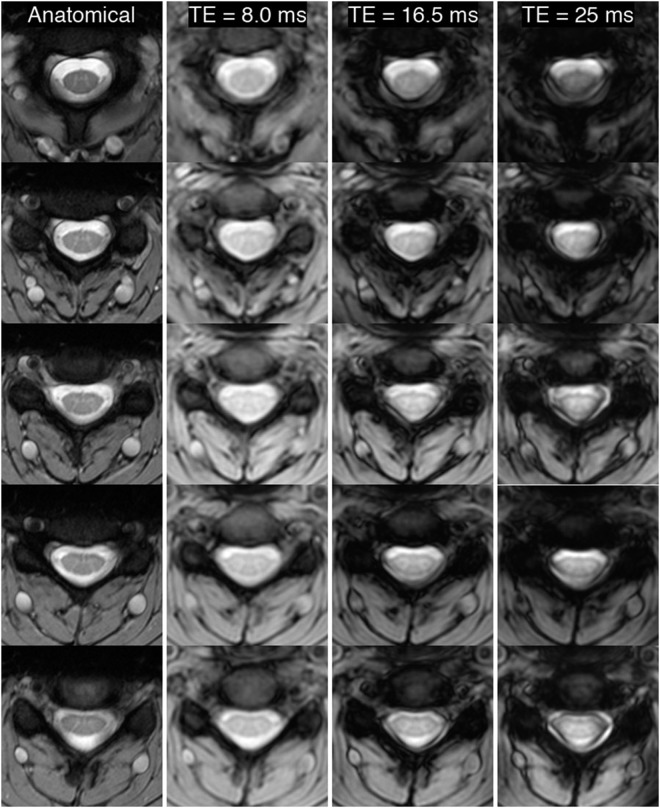
Visualization of five approximately equidistant anatomical slices (#1, #4, #6, #8, and #10, superior to inferior) and the corresponding registered mean functional slices with TE = 8.0, 16.5, and 25 ms in one representative subject. The grayscale values are kept constant across TEs by normalizing each image to its respective 98% percentile intensity. Across most slices in all subjects, longer TEs, especially at 25 ms, introduce significant artifacts that can significantly obscure spinal cord gray/white matter and increase the difficulty of accurate functional-to-anatomical registration. The corresponding TSNR maps for slices #2 to #11 are shown in Supp. Fig. S2.

To visualize the effects of increasing TE, Fig. 4 presents five equidistant anatomical slices (first column) from one subject, and the corresponding mean slices from functional runs with TE = 8.0, 16.5, and 25 ms. Qualitatively, functional images with TE = 8.0 ms have slightly lower contrast between gray and white matter, but also minimal artifacts and excellent geometric fidelity in the shape of the spinal cord and surrounding CSF. At TE = 16.5 ms, artifacts that overlap with CSF and impinge upon white matter structures appear on many slices. Finally, at TE = 25 ms, artifacts manifest on most slices, and overlap with CSF and white matter – and even impinge upon dorsal gray matter in a few slices. Such geometric artifacts may impact the accuracy of functional connectivity measurements by slightly distorting structures of interest (e.g., dorsal gray matter horns), and also by introducing functional-to-anatomical registration inaccuracies. The corresponding TSNR maps for these functional data are shown in Supp. Fig. S2.

**Figure 5:**
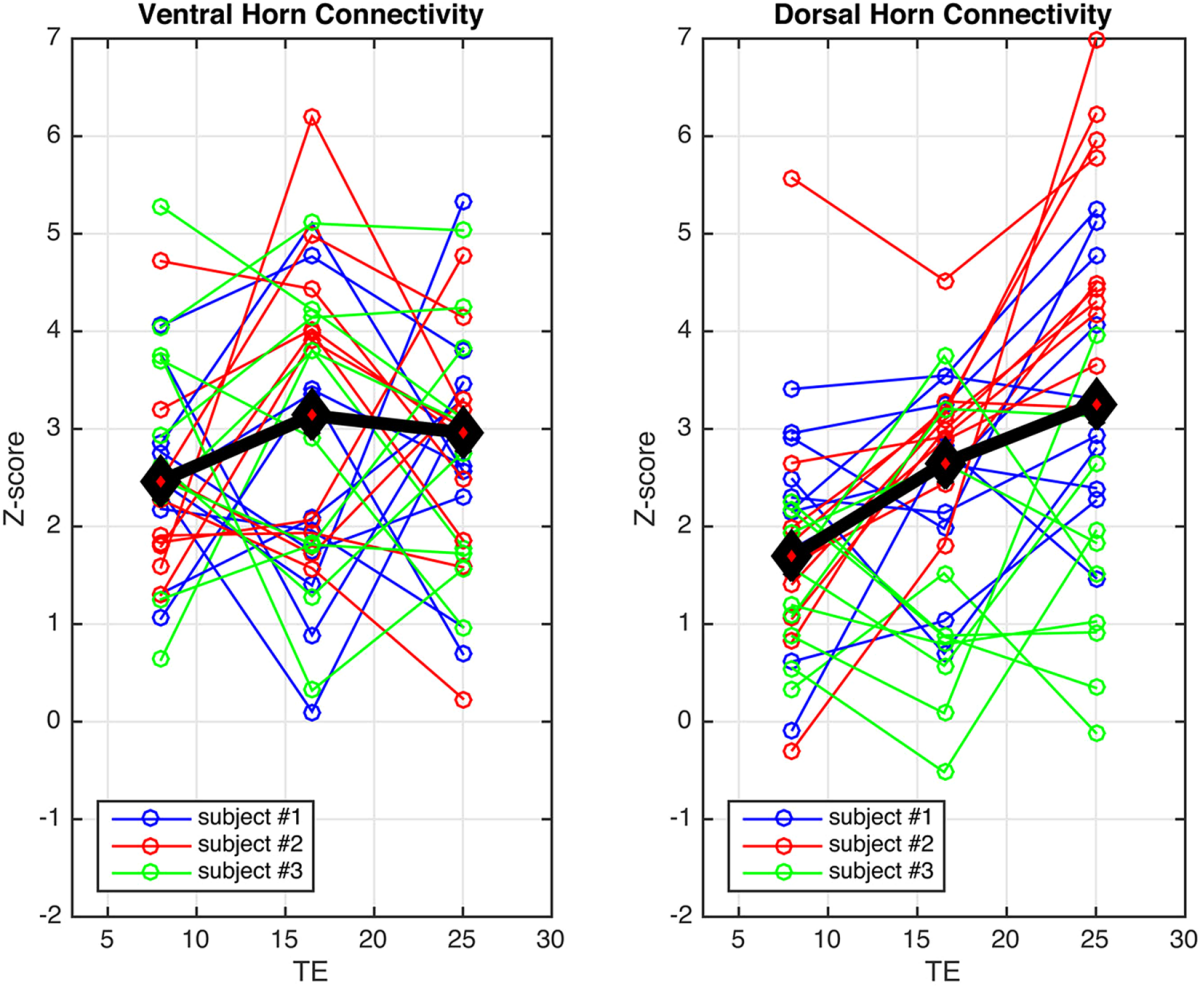
Measurements of connectivity between ventral horns and between dorsal horns for TE = 8.0, 16.5, and 25 ms, respectively, for three subjects. Based upon the results of the “scan length” study, a high-pass filter is used to retain all frequencies above 0.01 Hz. There are no statistically significant effects of TE on ventral *z*-scores for any subject, but dorsal *z*-scores are significantly higher at TE = 25 ms compared to TE = 8.0 ms for subject #1 (paired, *p* < 0.05) and subject #2 (paired, *p* < 0.001). A black and red diamond marks the median *z*-score for each aggregate column.

Figure 5 displays aggregate *z*-score plots across slices and subjects for functional runs acquired with TE = 8.0, 16.5, and 25 ms, respectively. After identical data-driven de-noising procedures, gray matter TSNR across subjects was 32.6 ± 4.3, 22.9 ± 5.3, and 16.1 ± 5.2, respectively, for data acquired with TE = 8.0, 16.5, and 25 ms. There are no statistically significant differences in ventral *z*-scores across TEs for any subject. For dorsal horn connectivity, there are no significant differences in *z*-scores between TE = 8.0 ms and 16.5 ms for any subject, but *z*-scores at TE = 25 ms are significantly higher than at TE = 8.0 ms for subject #1 (paired, *p* < 0.05; blue) and subject #2 (paired, *p* < 0.001; red), but not subject #3 (green). These results suggest that dorsal horn connectivity may be influenced by the choice of TE, and also support the notion that TSNR alone is a poor predictor of resting state network fidelity^38^.

## Discussion

This paper explores the impact of three factors – run length, bandpass filter width, and TE – on the detectability of resting state spinal cord networks. In one study, 20-min functional runs with a maximum VAT of 2.76 s were acquired to investigate frequencies up to 0.17 Hz. This duration was selected to investigate temporal correlations over what may be considered the longest feasible functional run to be performed during a clinical protocol. In another study, functional data were acquired with three TEs between 8.0 ms and 25 ms to similarly investigate network detectability, as well as qualitatively evaluate the manifestation of undesirable artifacts.

The conventional wisdom in brain fMRI is to set TE to be equal to T_2_* to maximize BOLD CNR. However, a review of the 4T study that established this recommendation shows that BOLD CNR vs. TE in tissue is a broad curve, and setting TE to be less than half of T_2_* resulted in only a ~15% decrease in BOLD CNR relative to the maximal value (Fig. 3c in^28^). A follow-up study similarly showed a broad curve for BOLD CNR in tissue and demonstrated that TE ≈ T_2_*/3 resulted in only a slight decrease in CNR at 4T (Fig. 3c in^17^). Therefore, although BOLD CNR may indeed be maximal when TE = T_2_*, in practice this is not a strict requirement and in fact is likely detrimental for some fMRI studies (e.g., at 7T and/or in the spinal cord) because maximizing BOLD CNR also maximizes signal dropout and geometric distortions.

The “scan length” study revealed that the most important factor in network detectability is the range of frequencies considered. For either acquisition strategy, “full-k” or “part-k”, increasing the upper frequency cut-off beyond the conventional 0.08 Hz resulted in higher *z*-scores. These data suggest that an 8-min run should be appropriate for detecting the ventral (motor) network at 3T if a higher frequency cut-off is used, but increasing the run length to 12 mins, if feasible, would be additionally advantageous. Furthermore, the “variable TE” study did not detect a significant effect of TE on ventral *z*-scores, and also showed that shorter TEs can obviate distortions and signal dropout on and around the spinal cord. Thus, if a spinal cord study was only interested in investigating the motor network, then these data suggest that selecting any value for TE over a broad range may be appropriate.

Reliable detection of the dorsal (sensory) network is, however, a more complicated story. The “scan length” study revealed dorsal *z*-scores to be approximately one-third lower than ventral *z*-scores, suggesting that longer runs may be needed to reliably detect the sensory network. Additionally, the “variable TE” study suggests that dorsal network detectability may increase if TE is lengthened from 8.0 ms to 25 ms – which may be seen as somewhat counterintuitive because the imaging artifacts also increase noticeably when TE = 25 ms. Importantly, Fig. 4 shows that artifacts at the longest TE manifest around the gray matter butterfly and encroach upon it, but impinge upon the dorsal horns in only a few slices. These artifacts clearly influence the accuracy of the functional-to-anatomical registration to some degree, but our custom registration algorithm was developed using 7T data and has been observed to work well even when functional images are slightly warped and/or contaminated by such artifacts. Therefore, a possible explanation for the results presented in Fig. 5 (right panel) is that the analyses of dorsal horn connectivity at TE = 25 ms still benefit from greater BOLD contrast because the artifacts impact white matter more than gray matter, and the custom registration algorithm is sufficiently robust to the deleterious influence of these artifacts to ensure that there is a reliable overlap between functional and anatomical images in nearly all slices.

The following paragraphs present five plausible (and not mutually exclusive) reasons that may, in part, explain why the data presented in the “scan length” study show that dorsal *z*-scores are generally lower than ventral *z*-scores.

First, though the ventral and dorsal networks are only ~1 cm apart, the manifestation of coherent bilateral dorsal fluctuations may intrinsically differ in one or more ways from its ventral neighbor. One possibility, as suggested by Fig. 5, is that the ventral and dorsal networks may follow different BOLD CNR vs. TE response curves. This difference would likely not be noticeable for TE ≈ T_2_* acquisitions, but would be noticeable when operating in a lower-TE regime. At 7T, T_2_* in spinal cord gray matter has been calculated to be 29.3 ± 4.5 ms^39^. Our 7T studies^12,13^ set TE = 7.8–8.0 ms, approximately T_2_*/3.6, and observed robust connectivity between both ventral and dorsal horns, though dorsal *z*-scores were lower. In comparison, the recent 3T work by Eippert *et al*. used a TE of 44 ms^21^ and did not report a clear divergence between the manifestation of the ventral and dorsal networks. If T_2_* of spinal cord gray matter at 3T is approximately 40 ms, then the use of TE = 8.0 ms for the “scan length” study herein is ~T_2_*/5 and, though significantly reducing geometric distortions and signal dropout, may be too low to reliably detect the dorsal (but, interestingly, not ventral; Fig. 3) network. This may serve to further support the translation of spinal cord MRI to 7T^40^ where fMRI benefits from increased BOLD CNR at higher fields.

Second, the manifestation of these resting state networks may also differ depending upon the cord segment examined. While the 3T studies herein and our previous 7T studies focused on higher cervical segments C2 to C5, Eippert *et al*. investigated lower segments C6 to T1^21^. The within-segment results presented in Eippert *et al*. (Fig. 4 and Table 1 in^21^) reveal comparable mean correlations between ventral horns (*r* = 0.10–0.17) and between dorsal horns (*r* = 0.11–0.16) for C7 to T1, but at C6 the mean correlation between ventral horns (*r* = 0.26) was quite a bit higher than dorsal horns (*r* = 0.19). It is possible that the diverging correlations at C6 represent the start of a trend that could explain the differences observed in upper-cervical segments, but a rigorous study of connectivity along the entire cervical cord would be needed to investigate this theory.

Third, while the “scan length” study demonstrated ventral *z*-scores that generally increased over the full 20 mins, dorsal *z*-scores initially increased only modestly, and then were relatively asymptotic after ~10 mins. Doubling the resting state run length from 10 to 20 mins without achieving even a modest increase in *z*-scores is a very curious observation, and hints that other factors, e.g., non-stationarity^41^, may be affecting the detectability of the dorsal network. Investigating the non-stationarity of functional connectivity in the brain is a complex topic^42^ that is still relatively new, and non-stationarity in spinal cord signaling is a plausible hypothesis that remains to be tested.

Fourth, it is important to also recognize that the dorsal horns are roughly half the thickness of the ventrolateral horns, and on the order of ~1–2 mm across. Therefore, with an in-plane functional resolution of 1 × 1 mm^2^, partial volume effects will impact the dorsal horns to a greater degree than the ventral horns, and measurements of dorsal gray matter connectivity, even after slight erosion of the interpolated high-resolution mask, will be impacted by adjacent white matter. Sub-millimeter spinal cord fMRI will thus be needed to further mitigate the undesirable influence of partial volume effects.

Finally, another possibility involves the approach by which these regions of interest (ROIs) are selected for the group analyses. As described in the Methods, gray matter masks were defined for each horn using the anatomical image, and then these masks were eroded slightly to minimize gray/white matter partial volume effects and produce the final ROIs used in these analyses (Supp. Fig. S1). This approach is appropriate because spinal cord gray matter is clearly visualized in the averaged multi-echo gradient echo anatomical images (Fig. 1B), and creates gray matter ROIs that are in the approximate center of each horn. However, slight geometric distortions on the order of ~1 mm or less would prevent an anatomical ROI from overlapping with the underlying functional data as intended. Examples of this may be seen by comparing the anatomical image in Fig. 1B with its corresponding functional image in Fig. 1C, or noting that the seed region manually selected in Fig. 3 is more medial than the approximate center of the ventral gray matter horn. Additionally, while we posit that the foci of correlated BOLD signal changes will manifest in the center of the gray matter horns, which appears to be a reasonable approach for the ventral horns in many slices, there does not appear to be strong evidence that the same approach is appropriate for the dorsal horns. Perhaps coherent BOLD signal fluctuations in the dorsal horns, which are long and narrow, tend to manifest closer to the sensory nerve root? This is not yet known. Overall, the culmination of these factors suggest that defining gray matter ROIs on the anatomical images alone may not be optimal, and that reliably detecting the true foci for dorsal (and ventral) networks may require a more data-driven approach, possibly also with a probabilistic atlas^43^, that considers the spatial contrast and features of anatomical and functional images in tandem. Defining ROIs using a probabilistic atlas^43^ is an approach that has been recently used by other groups^21,23^, and could have also been implemented herein if our high-resolution anatomical images did not have sufficient gray/white matter contrast (e.g., possibly due to T_2_-weighting) to resolve the small features of the gray matter horns.

The implementation of two acquisition sequences in the “scan length” study – “full-k” and “part-k”, respectively – also permits an evaluation of the potential benefits of using a partial Fourier scheme to decrease the VAT from 2.76 s to 2.08 s per volume, thereby increasing the number of volumes acquired from 434 to 580 for a 20-min scan. This partial Fourier encoding is, however, achieved at the expense of a ~11% decrease in TSNR plus the loss of image phase information after partial Fourier reconstruction, so it is not clear *a priori* if the faster sampling rate translates into an increase in detectability (i.e., significantly higher *z*-scores). In Fig. 2, the “top-tier” curves with the highest *z*-scores are blue [VAT = 2.76 s and BW = 0.01–0.13 Hz], yellow [VAT = 2.76 s and BW = 0.01–0.17 Hz], and magenta [VAT = 2.08 s and BW = 0.01–0.17 Hz]. These three curves represent the highest group *z*-scores achievable by each acquisition sequence. Importantly, these curves visually overlap in the analyses of both ventral and dorsal connectivity, and, as such, the absence of any significant difference between the blue/yellow (VAT = 2.76 s) and magenta (VAT = 2.08 s) curves does not show that one acquisition sequence is preferable over the other.

A subtle but noteworthy difference between these 3T studies and our 7T reproducibility study^13^ is the approach by which the metric of spinal cord connectivity is calculated. The approach presented in our 7T work considered a vector of single-voxel correlations, and then selected the 95^th^ percentile of this *z*-score vector as a conservative metric that protected against potential spuriously high single-voxel correlations. We initially used the same approach to analyze our 3T data, but, upon closer inspection of this vector, did not observe much evidence of spuriously high correlation values that would bias the results significantly upwards and thus need to be avoided. At 3T, selecting the 95% percentile instead of the maximum value just shifted the correlations down slightly, and so we felt that it was appropriate to select the maximum of the correlation vector as the metric of functional connectivity for the studies herein.

While this paper has explored considerations that are common in the development of any fMRI protocol, two limitations may be addressed in future studies. First, while the “scan length” study analyzed 100 aggregate data points per time point (Fig. 2), and the “variable TE” study presents 30 data points per TE (Fig. 5), both studies could have benefited from acquiring data from an expanded cohort of healthy volunteers. Specifically, while the results of the “variable TE” study show a significant difference in dorsal *z*-scores between TE = 8.0 ms and TE = 25 ms for two of the three subjects, we consider these results to be interesting yet still preliminary, and a follow-up study with a larger cohort of subjects is required to further investigate possible effects of TE on dorsal horn connectivity. Second, while we investigated the relative optimization of 3D acquisition sequences, motivated by our previous 7T work showing that such sequences are helpful in mitigating geometric distortions in both the brain^44^ and spinal cord^12,13,27^, it is important to note that 2D EPI is still commonly used for 3T fMRI, and future studies should also explore similar optimizations of 2D EPI sequences. Methodological studies that aim to optimize one or more components of the fMRI acquisition/processing/analysis pipeline have been conducted in the brain since the inception of BOLD fMRI, and so, for the cervical cord, the studies presented herein may serve as a starting point to facilitate the development of general recommendations for practical spinal cord fMRI.

In summary, this report has investigated the effects of run length, bandpass filter width, and TE on the detectability of resting state spinal cord networks. Firstly, the results herein provide strong evidence that the upper frequency cut-off for the bandpass filter should extend beyond the conventional 0.08 Hz that is commonly used in the brain. Secondly, a run length of ~8 mins should be sufficient for reliable detection of the ventral (motor) network, though a 12-min run, if feasible, would be advantageous. Thirdly, while ventral connectivity does not appear to exhibit a significant TE dependence for the range of TEs considered, a TE of ~T_2_*/1.6 may enhance the detection of the dorsal (sensory) network but also introduce geometric distortions and signal dropout that can be partially addressed through robust post-processing methods. At 3T, a TE of ~T_2_*/2.4 may be an acceptable compromise between BOLD sensitivity and mitigation of geometric distortions. The combined 3T and 7T evidence to date suggests that the manifestation of dorsal horn connectivity may differ from ventral horn connectivity, and further investigations are certainly warranted to more fully understand and appreciate the nature of resting state spinal cord networks in health and in disease.

## Methods

### Data acquisition

Experiments were performed on a Philips Achieva 3T scanner (Best, The Netherlands) with a dual-channel transmit body coil and the vendor’s 16-channel neurovascular coil (8 head elements combined into 6 channels, 4 neck channels, and 6 upper chest channels) for signal reception covering the brain and cervical cord. All volunteers were healthy with no history of spinal cord injury or neurological impairment. Subjects were recruited, provided informed consent, and were scanned under a protocol approved by the Vanderbilt University Institutional Review Board. All methods were performed in accordance with the relevant guidelines and regulations.

Each scanning session began with a sagittal localizer to identify the general anatomy and location of the C3/C4 intervertebral disc, and the imaging stack was centered on the C3/C4 disc so that all slices were, as best as possible, perpendicular to the cord (e.g., Fig. 1A). The imaging stack covered vertebral levels C2 to C5, roughly corresponding to spinal nerve root levels C3 to C6^45^.

High-resolution axial anatomical images were acquired using an averaged multi-echo gradient echo (mFFE)^46^ T_2_*-weighted sequence with the following parameters: field of view (FOV) = 150 × 150 mm, acquired voxel size = 0.65 × 0.65 × 5 mm^3^, interpolated voxel size = 0.29 × 0.29 × 5 mm^3^, 12 slices, slice thickness = 5 mm, first TE = 7.20 ms, 4 additional echoes where ΔTE = 8.83 ms (5 echoes in total), repetition time (TR) = 700 ms, flip angle = 28°, sensitivity encoding (SENSE)^47^ = 2.0 (left-right), and number of acquisitions averaged = 2. Total acquisition time = 5 mins and 26 s. A saturation band was positioned anterior to the spinal cord to suppress signal from the mouth and throat.

In the first fMRI study (“scan length”), ten subjects (5 male, 24–36 years; 5 female, 21–35 years; 27.0 ± 4.9 years) performed two consecutive 20-min resting state runs with a 3D gradient-echo sequence: FOV = 150 × 150 mm, voxel size = 1 × 1 × 5 mm^3^, 12 slices, slice thickness = 5 mm, TE = 8.0 ms, TR = 34 ms, flip angle = 8°, echo train length (ETL; k-space lines per radiofrequency pulse) = 7, and SENSE = 2.0 (left-right). To investigate the trade-off between TSNR and degrees of freedom in detecting correlated BOLD signal fluctuations, one fMRI sequence (“full-k”) did not employ a partial Fourier scheme and had a VAT of 2.76 s (434 volumes) whereas the other sequence (“part-k”) used partial Fourier factors of 0.714 in-plane and 0.8 through-plane to reduce the VAT to 2.08 s (580 volumes). The order of the two runs alternated between subjects.

In the second study (“variable TE”), three subjects (28.3 ± 5.8 years) each performed three 7.8-min resting state runs with similar 3D gradient-echo parameters: FOV = 150 × 150 mm, voxel size = 1 × 1 × 5 mm^3^, 12 5-mm slices, TR = 45 ms, flip angle = 10°, ETL = 7, SENSE = 2.0, and VAT = 3.66 s (128 volumes). A VAT of 3.66 s was the minimum achievable VAT (without partial Fourier encoding) for the longest TE acquisition, and was kept consistent across lower TE acquisitions. To investigate the potential trade-off between BOLD sensitivity and signal-dropout/TSNR, the runs had TE = 8.0 ms, 16.5 ms, or 25.0 ms, respectively, with the order permuted between subjects.

For all functional runs, respiratory and cardiac cycles were externally monitored and continuously recorded using a respiratory bellows and pulse oximeter, and 10 “dummy” volumes were acquired (and automatically discarded) to compensate for the approach to steady-state magnetization.

### Data processing

Functional data from each run were processed using the spinal cord pipeline previously described in detail^13^. In brief, each functional slice was processed to correct for motion and partially mitigate physiological noise sources extracted from the external recordings via RETROICOR^48^ (steps #1–#6 in^13^). Masks defining the boundaries of gray matter, white matter, and cerebrospinal fluid were manually created for each slice based upon the unique features of each high-resolution anatomical image (step #7 in^13^). Resultant functional images were then registered to their respective anatomical images (steps #8–#11 in^13^), and then further de-noised using slice-wise data-driven ‘regressors of no interest’ (steps #12 and #13 in^13^). In preparation for the group functional connectivity analyses (Figs 2 and 5), gray matter masks were subdivided into quadrants to identify left and right ventral and dorsal horns, and each sub-region was morphologically eroded slightly to mitigate partial volume effects and identify the approximate center of each ROI (step #15 in^13^). An example of the resultant ROIs for one subject is shown in Supp. Fig. S1. In the single-subject analysis performed in one subject (Fig. 3), a single voxel time course is used as the seed.

In the “scan length” study, group fMRI data were bandpass filtered between 0.01 Hz and 0.08, 0.13, or 0.17 Hz, respectively, using a Chebyshev Type II filter (‘cheby2’ and ‘filtfilt’ in Matlab). An upper limit of 0.17 Hz was selected because that value was close to the Nyquist frequency of “full-k” data. In the single-subject analysis, a bandpass filter with BW = 0.01–0.17 Hz was applied to the single voxel time course. Based upon the findings of the “scan length” study (described in the Results), as well as the observations by Eippert *et al*.^21^, the subsequent “variable TE” study used a high-pass filter to retain all frequencies above 0.01 Hz.

### Data analysis

Our initial 7T investigations explored temporal correlations between all possible combinations of gray and white matter ROIs^12^, and also calculated ventral-ventral, ventral-dorsal, and dorsal-dorsal horn connectivity using both full and partial correlations (where the partial correlation between two ROIs regressed out signal fluctuations from the adjacent two ROIs)^13^. The latter study reported relatively minor differences between full and partial correlations, and weak evidence of reproducible correlations between ventral and dorsal horns. Therefore, based upon these results, the 3T studies presented herein consider only full correlations between ventral and between dorsal horns. Full correlation is defined as the maximum *z*-score between pairs of voxels within ROIs on the same slice, and *z* is calculated using the Fisher *r*-to-*z* transformation *z* = *tanh*^−1^(*r*)(*dof* − 3)^1/2^ where *dof* is the estimated degrees of freedom after correction for first-order auto-correlation^49^.

Gray matter TSNR, a common proxy for overall data quality, was calculated for each voxel as the mean intensity across time divided by the standard deviation. The 3D slab excitation profile used to acquire the fMRI data generally resulted in lower TSNR on the first and last slices, so presented results were obtained from analyses performed on the innermost 10 slices to minimize potential data quality bias. Reported TSNR values are the median across gray matter voxels plus or minus the standard deviation.

Results from group studies (Figs 2 and 5, respectively) were analyzed in GraphPad Prism 6 (GraphPad Software, La Jolla, CA) using non-parametric Kruskal-Wallis tests (if unpaired) or Friedman tests (if paired); if significant, groups were then compared using Dunn’s post hoc (multiple comparisons) tests. Results with *p* < 0.05 after correction for multiple comparisons were viewed as statistically significant.

## Electronic supplementary material

Supplementary Information

## Data Availability

The de-identified MRI datasets acquired for this study are available from the corresponding author upon reasonable request.
