# Supplementary material for: A practical protocol for measurements of spinal cord functional connectivity
Source: Sci Rep. 2018 Nov 8;8:16512. doi: 10.1038/s41598-018-34841-6 (PMC6224587; doi:10.1038/s41598-018-34841-6)
Supplement: Supplementary file 1 — Supplementary Information [file 41598_2018_34841_MOESM1_ESM.pdf]

Supplementary Information for "A practical protocol for measurements of spinal cord functional connectivity"

Robert L. Barry[1,2,*], Benjamin N. Conrad[3,4], Seth A. Smith[3,5,6], and John C. Gore[3,5,6]

[1]Athinoula A. Martinos Center for Biomedical Imaging, Department of Radiology, Massachusetts General Hospital, Charlestown, Massachusetts, USA
[2]Department of Radiology, Harvard Medical School, Boston, Massachusetts, USA
[3]Vanderbilt University Institute of Imaging Science, Vanderbilt University Medical Center, Nashville, Tennessee, USA
[4]Neuroscience Graduate Program, Vanderbilt University Medical Center, Nashville, Tennessee, USA
[5]Department of Radiology and Radiological Sciences, Vanderbilt University Medical Center, Nashville, Tennessee, USA
[6]Department of Biomedical Engineering, Vanderbilt University, Nashville, Tennessee, USA
[*]Corresponding author (`Robert.Barry@mgh.Harvard.edu`)

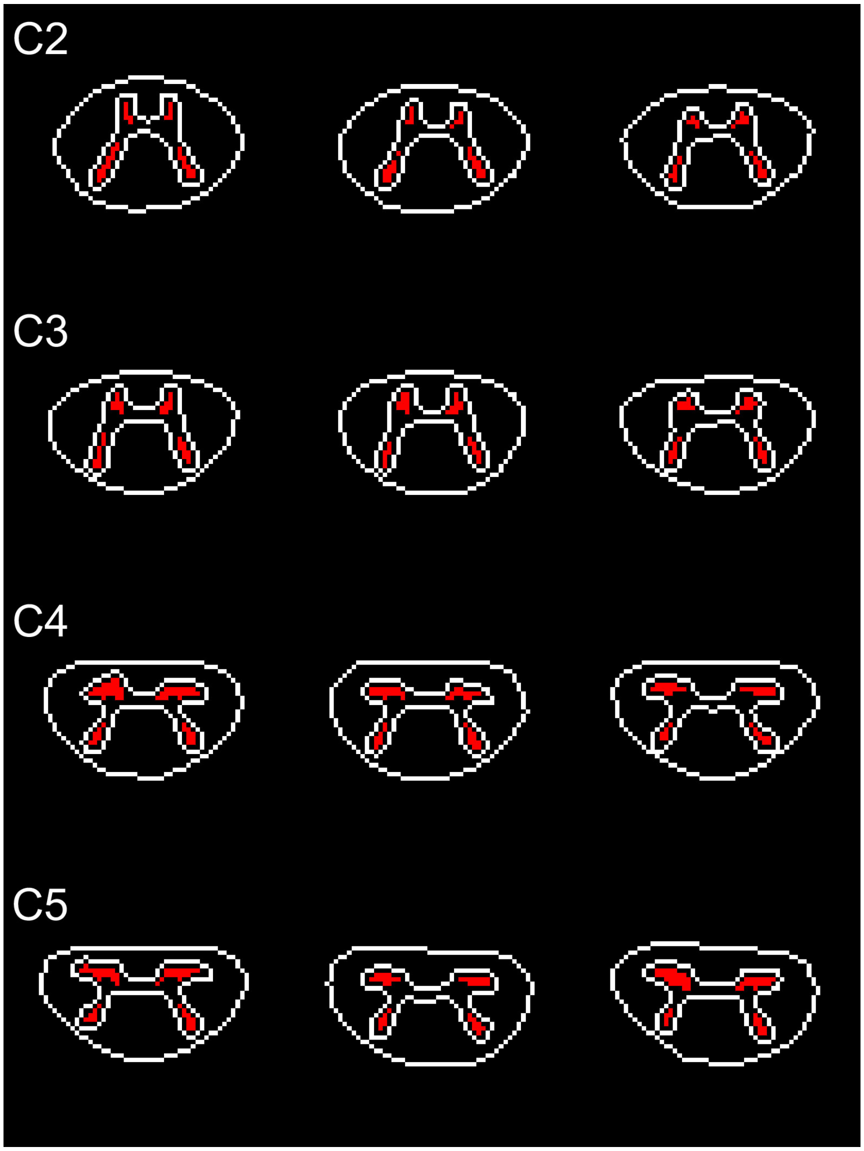

Figure S1: Outline of spinal cord white matter and gray matter (white) across vertebral levels in one subject, and the corresponding eroded gray matter ROIs (red) used in the within-slice analyses of functional connectivity.

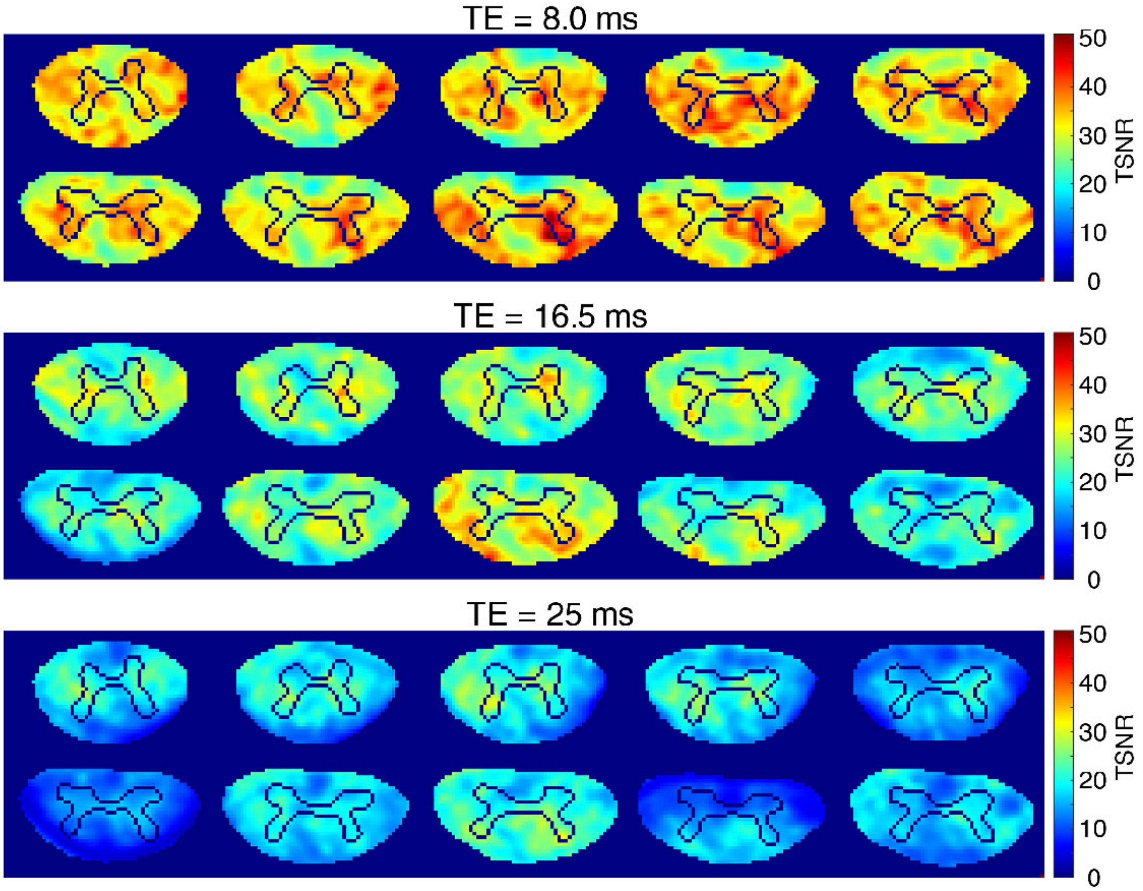

Figure S2: Temporal signal-to-noise ratio (TSNR) maps for the fMRI data shown in Fig. 4. For each TE, the middle 10 slices (from C2 to C5) used in the analyses of functional connectivity are displayed. For this subject (subject #1 with respect to the data presented in Fig. 5), gray matter TSNR is 32.3±4.7, 23.6±4.7, and 15.9±4.9, respectively, for data acquired with TE = 8.0, 16.5, and 25 ms.

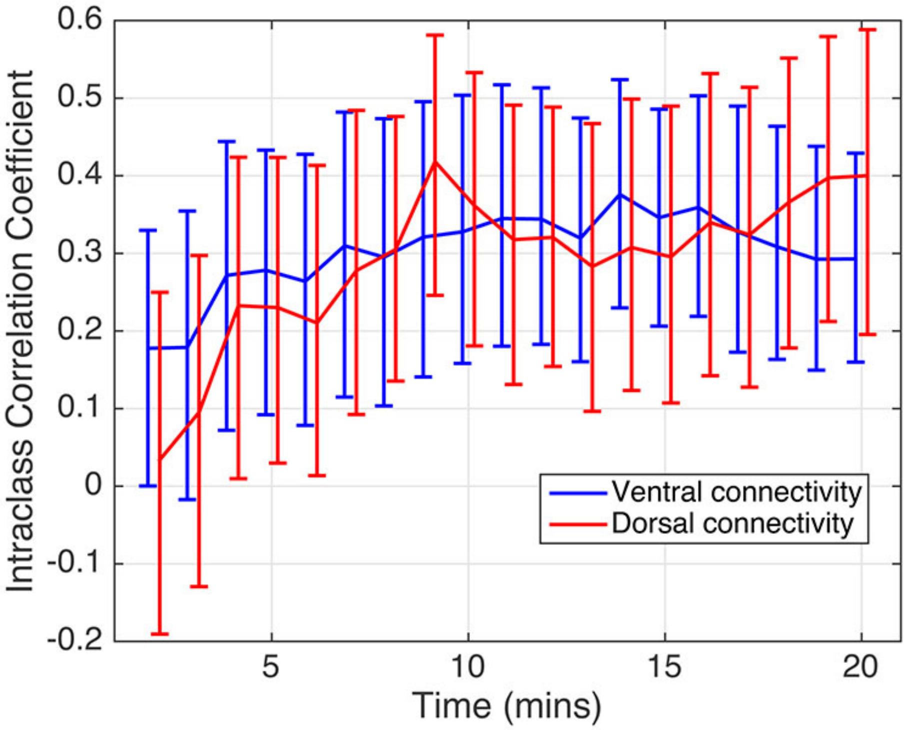

Figure S3: Trajectory of intraclass correlation coefficient (ICC) as a function of time in the "scan length" study. A high-pass filter is used to retain all frequencies above 0.01 Hz. At each min between $t = 2$ and $t = 20$ mins, 1) z-scores are calculated for ventral horn and dorsal horn connectivity in the "full-k" and "part-k" runs, respectively, and 2) the 'Case 2' ICC [36] is calculated between runs using the 100 aggregate z-scores across slices and subjects. The 95% confidence interval (C.I.) for each ICC, denoted by errors bars, is estimated using a bootstrap procedure ('bootci' in Matlab) and 50,000 subsamples generated using random sampling with replacement. The curves for ventral (blue) and dorsal (red) connectivity are temporally offset from one another slightly to better visualize the error bars. Due to the wide and significantly overlapping 95% C.I. at each time point, no statistical tests are performed on these data. At $t = 8$ mins, the ICC for ventral connectivity is 0.30 (95% C.I. = (0.10, 0.47)) and the ICC for dorsal connectivity is 0.31 (95% C.I. = (0.14, 0.48)). In comparison, the ICCs for ventral and dorsal connectivity at 7T, calculated between two consecutive 8.35-min runs using similar analysis methods (Supp. Figs. S2A and S2B in [13]), were 0.58 (95% C.I. = (0.48, 0.66)) and 0.56 (95% C.I. = (0.46, 0.65)), respectively. The scatter plots used to generate the ICCs at one time point, $t = 10$ mins, are shown in Supp. Fig. S4.

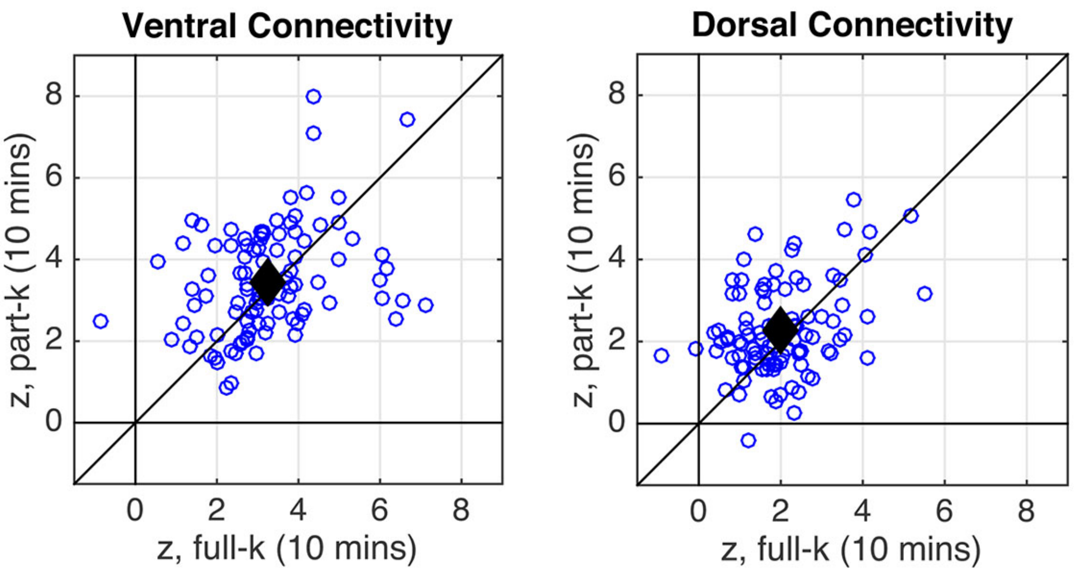

Figure S4: Scatter plots of ventral (left) and dorsal (right) z-scores across all slices and subjects, calculated using only the first 10 mins of the "full-k" and "part-k" runs. A high-pass filter is used to retain all frequencies above 0.01 Hz. In each plot, the black diamond represents the center of mass of the 100 correlation pairs. The ICCs for these data, shown in Supp. Fig. S3 at $t = 10$ mins, are 0.33 (95% C.I. = (0.16, 0.50)) for ventral connectivity and 0.36 (95% C.I. = (0.18, 0.53)) for dorsal connectivity.

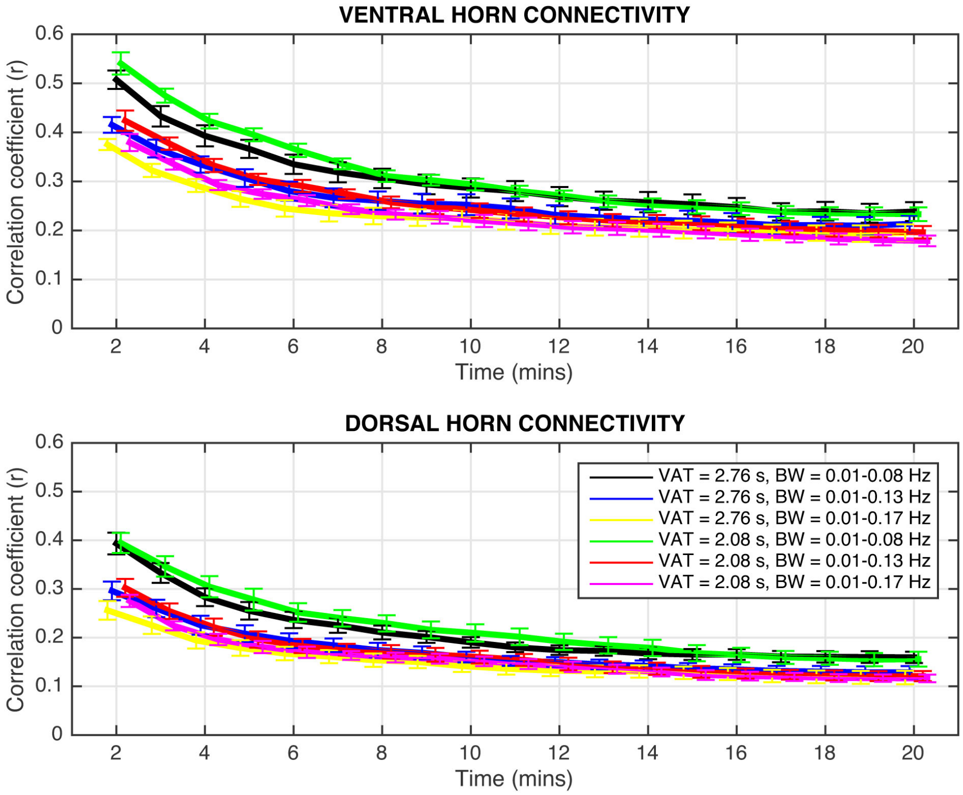

Figure S5: Pearson correlation coefficient (r) between ventral horns (top) and dorsal horns (bottom) for the data presented in Fig. 2. Only points at every minute are displayed, and, for clarity, the curves are temporally offset from one another slightly to better visualize the error bars. Error bars represents standard error of the mean across subjects. In the bottom panel, the figure legend relating color to volume acquisition time (VAT) and bandwidth (BW) refers to curves in both top and bottom panels.

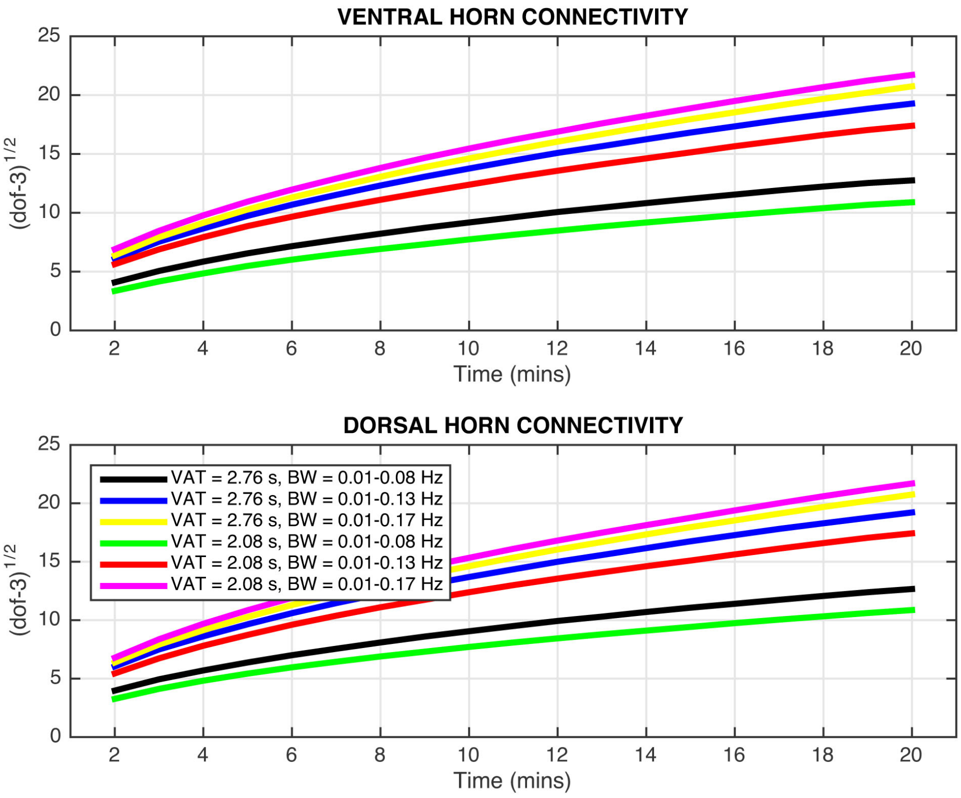

Figure S6: Multiplicative term $(dof-3)^{1/2}$ from the Fisher r-to-z transformation used to calculate z-scores between ventral horns (top) and dorsal horns (bottom) for the data presented in Fig. 2. Only points at every minute are displayed. In the bottom panel, the figure legend relating color to volume acquisition time (VAT) and bandwidth (BW) refers to curves in both top and bottom panels.

Table S1: Summary of statistically significant comparisons presented in the Results.

"scan length" study

comparison performed	statistical result
Ventral horns at $t = 8$ mins: z-scores with [VAT = 2.76 s and BW = 0.01–0.13 Hz] (blue curve) are higher than z-scores with [VAT = 2.08 s and BW = 0.01–0.08 Hz] (green curve)	$p < 0.05$, unpaired
Ventral horns at $t = 8$ mins: z-scores with [VAT = 2.08 s and BW = 0.01–0.17 Hz] (magenta curve) are higher than z-scores with [VAT = 2.08 s and BW = 0.01–0.08 Hz] (green curve)	$p < 0.01$, paired
Dorsal horns at $t = 8$ mins: z-scores with [VAT = 2.08 s and BW = 0.01–0.13 Hz] (red curve) are higher than z-scores with [VAT = 2.08 s and BW = 0.01–0.08 Hz] (green curve)	$p < 0.05$, paired
Dorsal horns at $t = 8$ mins: z-scores with [VAT = 2.08 s and BW = 0.01–0.17 Hz] (magenta curve) are higher than z-scores with [VAT = 2.08 s and BW = 0.01–0.08 Hz] (green curve)	$p < 0.05$, paired
Ventral horns with [VAT = 2.08 s and BW = 0.01–0.17 Hz] (magenta curve) between $t = 8$ and $t = 12$ mins: z-scores are higher at $t = 11$ mins than at $t = 8$ mins	$p < 0.05$, paired
Ventral horns with [VAT = 2.08 s and BW = 0.01–0.17 Hz] (magenta curve) between $t = 8$ and $t = 12$ mins: z-scores are higher at $t = 12$ mins than at $t = 8$ mins	$p < 0.001$, paired
Dorsal horns with [VAT = 2.08 s and BW = 0.01–0.17 Hz] (magenta curve) at $t = 13$–20 mins vs. $t = 8$ mins: z-scores are higher at $t \geq 18$ mins than at $t = 8$ mins	$p < 0.05$, paired

"variable TE" study

comparison performed	statistical result
Dorsal horns for subject #1 (blue): z-scores are higher with TE = 25 ms than TE = 8.0 ms	$p < 0.05$, paired
Dorsal horns for subject #2 (red): z-scores are higher with TE = 25 ms than TE = 8.0 ms	$p < 0.001$, paired
